# Incomplete Atypical Femoral Fracture Treated by Prophylactic Intramedullary Nail Fixation: A Case Series

**DOI:** 10.7759/cureus.22725

**Published:** 2022-02-28

**Authors:** Tomohiko Sakuda, Osamu Omoto, Takahiko Hamasaki, Nobukazu Okimoto, Nobuo Adachi

**Affiliations:** 1 Orthopaedics, Department of Orthopaedic Surgery, Graduate School of Biomedical and Health Sciences, Hiroshima University, Hiroshima, JPN; 2 Orthopaedics, Department of Orthopaedic Surgery, Saiseikai Kure Hospital, Kure, JPN; 3 Orthopaedics, Department of Orthopaedic Surgery, National Hospital Organization Kure Medical Center and Chugoku Cancer Center, Kure, JPN; 4 Orthopaedics, Department of Orthopaedic Surgery, Okimoto Clinic, Kure, JPN

**Keywords:** teriparatide, prophylactic fixation, osteoporosis, bisphosphonate, atypical femoral fracture

## Abstract

Long-term bisphosphonate use may be associated with atypical femoral fractures. In this report, we describe three cases of bisphosphonate-associated incomplete atypical femoral fracture, treated by prophylactic intramedullary nail fixation. Patients with long-term intake of bisphosphonates must be carefully monitored; atypical femoral fracture should be suspected in the presence of symptoms such as thigh pain. Its early identification is important to avoid a complete fracture and invasive surgery, and prophylactic fixation is recommended for incomplete atypical femoral fractures.

## Introduction

Bisphosphonates inhibit bone resorption, increase bone density, and help prevent fractures, and are therefore widely used for the treatment of osteoporosis. However, bisphosphonates may also severely suppress bone turnover [1], resulting in secondary calcification and accumulated microdamage which weaken the bone [2]. Thus, long-term bisphosphonate use may be associated with atypical femoral fractures. Atypical femoral fractures were first described by Odvina et al. in 2005 [1]. These occur non-traumatically or due to minor traumatic injury and are usually seen in the area from below the trochanter to the shaft. Cortical bone hypertrophy is normally evident, and the fracture appears from the external side as a transverse or simple oblique fracture with no comminution at the fracture site.

Surgical treatment is recommended for complete femoral fractures, but treatment of incomplete atypical femoral fractures is controversial [3]. The restriction of activities of daily life with partial weight bearing may be an option, but it does not appear to be reliable because of the risk of progression to a complete fracture. Early diagnosis and prophylactic fixation may allow patients to achieve early weight bearing and recovery. The use of bisphosphonates, however, may delay the healing of bisphosphonate-associated incomplete atypical femoral fractures. We hypothesize that improving bone metabolism by stimulating bone formation with drugs such as teriparatide may encourage fracture healing. Teriparatide has been shown to promote and even accelerate fracture healing in both animal models [4] and humans [5].

In this report, we describe three cases of bisphosphonate-associated incomplete atypical femoral fractures treated by prophylactic intramedullary nail fixation with and without teriparatide administration.

## Case presentation

The patients described below have given permission for the details of their cases to be presented for publication.

Case 1

**Figure 1:**
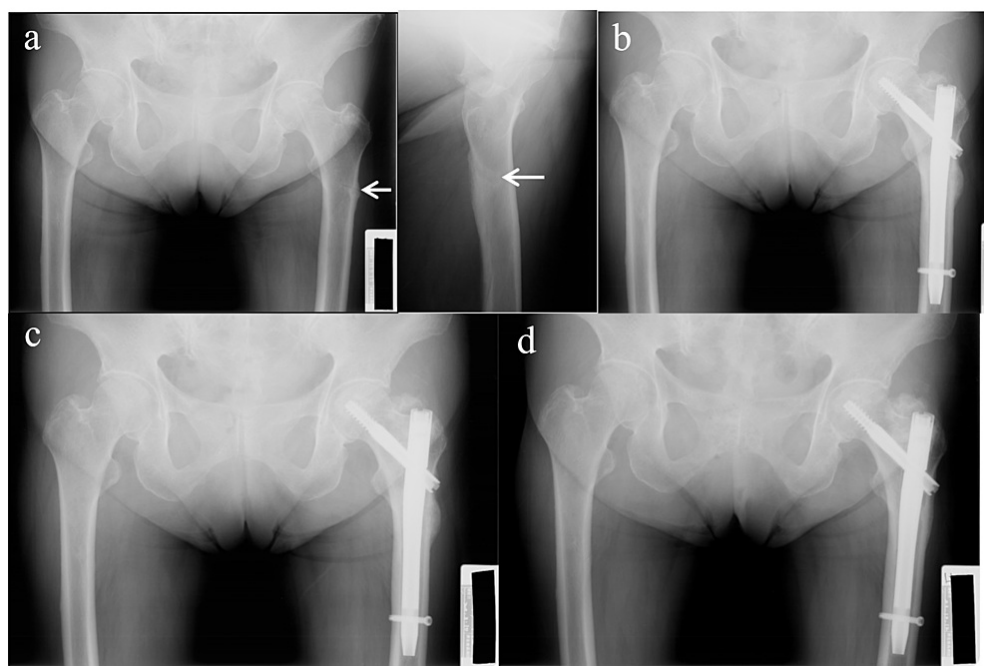
Case 1: Left subtrochanteric fracture (a) Initial examination (AP view and axial view). (b) Immediately after surgery. (c) 6 months postoperatively. (d) 15 months postoperatively.

A 72-year-old woman underwent left high tibial osteotomy and right total knee arthroplasty (TKA) due to knee osteoarthritis at another hospital. She has been taking alendronate (35 mg/week orally) for the treatment of osteoporosis for 41 months. While walking, she experienced sudden unexplained pain in the left thigh that left her unable to walk, and she was transported to our department by ambulance that day. Initial radiography revealed an incomplete subtrochanteric fracture of the left femur with lateral cortical bone hypertrophy, with the appearance of a stress fracture (Figure 1A). Dual-energy X-ray absorptiometry (DEXA) at the lumbar region showed a T-score of 0.73 and a bone mineral density (BMD) of 1.208 g/cm2, while the right femoral neck had a T-score of −2.67 and BMD of 0.738 g/cm2. Internal fixation using a proximal femoral nail was performed to achieve early weight bearing and recovery (Figure 1B). Full weight bearing on the affected leg was permitted on the day after the surgery, and alendronate was discontinued. The fracture line was still visible on radiography six months postoperatively (Figure 1C), but at 15 months postoperatively, bone union was complete and the hypertrophic bone had also disappeared (Figure 1D).

Case 2

**Figure 2:**
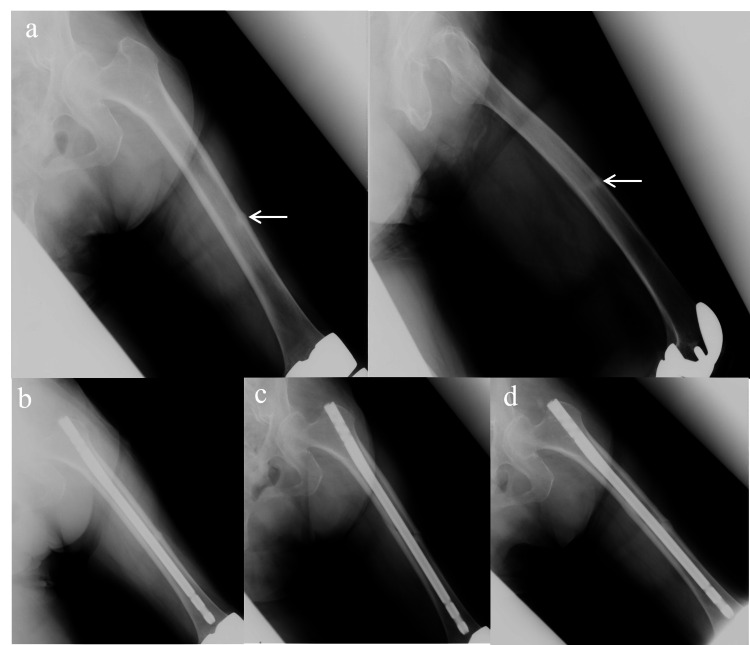
Case 2: Left femoral shaft fracture (a) Initial examination (AP view and lateral view). (b) Immediately after surgery. (c) 2 months postoperatively. (d) 6 months postoperatively.

An 88-year-old woman had undergone bilateral TKA in our department and had been taking alendronate (35 mg/week orally) for the past 50 months for osteoporosis. She experienced unexplained pain in the left thigh and weakness of the left leg but was ambulatory at our outpatient unit. An initial radiograph revealed an incomplete fracture of the left femoral shaft with lateral cortical bone hypertrophy (Figure 2A). On DEXA, the lumbar region had a T-score of −2.01 and BMD of 0.839 g/cm2, while the right femoral neck had a T-score of −5.94 and BMD of 0.452 g/cm2. Laboratory tests for bone metabolic markers showed that the level of the bone formation marker serum type I procollagen N-terminal pro-peptide (P1NP) was 28.9 μg/l (reference range 14.9-68.8 μg/l), while the level of the bone resorption marker tartrate-resistant acid phosphatase isoform 5b (TRACP-5b) was 315 mU/dl (120-420 mU/dl). Atypical femoral fracture was diagnosed, and alendronate was preoperatively discontinued. We performed internal fixation using a proximal femoral nail (Figure 2B). Full weight bearing on the affected leg was permitted on the day after the surgery, and weekly teriparatide subcutaneous injections (Teribone®, Asahi Kasei Pharma, Tokyo, Japan) at a dose of 56.5 μg were started on the same day. The fracture line was still visible at two months postoperatively (Figure 2C), but at six months postoperatively, bone union was complete and the hypertrophic bone had also disappeared (Figure 2D).

Case 3

**Figure 3:**
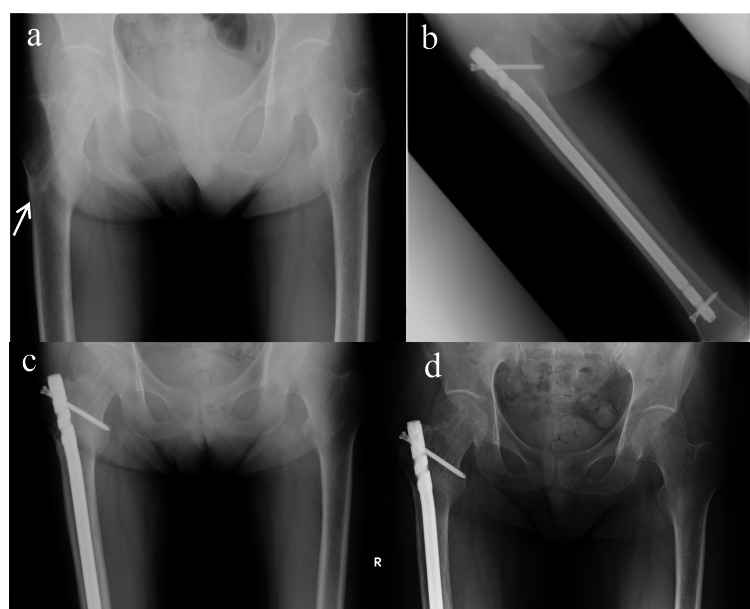
Case 3: Right subtrochanteric fracture (a) Initial examination. (b) Immediately after surgery. (c) 1 month postoperatively. (d) 5 months postoperatively.

A 47-year-old man had been taking desmopressin, levothyroxine, and prednisolone (at a dose of 15-20 mg/day) for panhypopituitarism for approximately 30 years. He had suffered a right femoral neck fracture in a fall four years previously, which was fixed with pins. He was started on alendronate at a dose of 35 mg/week orally postoperatively for osteoporosis, and the pins were removed 41 months later. Six months after removal, he experienced unexplained pain in the right thigh, but was ambulatory at the outpatient clinic. Initial radiography revealed a fracture line and lateral cortical bone hypertrophy peripheral to the insertion point of the pin (Figure 3A). On DEXA, the T-score and BMD of the lumbar region were −3.9 and 0.583 g/cm2, respectively and those of the left femoral neck were −3.7 and 0.399 g/cm2, respectively. Laboratory tests for bone metabolic markers showed a low level of P1NP (13.3 μg/l) and a TRACP-5b of 231 mU/dl. Atypical femoral fracture was diagnosed, alendronate was preoperatively discontinued, and internal fixation using a proximal femoral nail were performed (Figure 3B). Full weight bearing on the affected leg was permitted on the day after the surgery. The following week, 20 μg teriparatide subcutaneous injections (Forteo®, Eli Lilly and Company, Indianapolis, IN, USA) were started and administered once daily. The fracture line was still visible on radiography one month postoperatively (Figure 3C), but by five months postoperatively, bone union was complete and the hypertrophic bone had also disappeared (Figure 3D). Bone metabolic marker levels rose (P1NP: 74.4 μg/l, TRACP-5b: 363 mU/dl) after a year of daily teriparatide injections.

## Discussion

All three patients were taking bisphosphonates (alendronate 35 mg/week) for at least three years (Case 1 for 41 months, Case 2 for 50 months, and Case 3 for 41 months), which may have contributed to the development of atypical femoral fracture. A few reports have discussed epidemiological data regarding the association between bisphosphonates and atypical femoral fracture. Some studies have found significant differences in incidence between patients taking bisphosphonates for less than two years versus those taking them for eight years or more [6]; the incidence reportedly rises when bisphosphonates are used for five years or more [7]. Furthermore, 45.4% of patients taking bisphosphonates who developed atypical femoral fracture had been using them for three years or more [8].

Long-term glucocorticoid use is said to be another significant risk factor for atypical femoral fracture [9]. In Case 3, both bisphosphonates and glucocorticoids were involved in the severe suppression of bone turnover, and both bone resorption and formation were markedly suppressed. Another cause of atypical femoral fracture is a bowed femoral shaft [10,11], but this was not seen in any of our three cases.

Considering these previous reports and our own experience, we carry out regular check-ups on patients who have been taking bisphosphonates for three years or more, while monitoring their bone metabolic markers and bone mineral density. They are followed up with femoral X-rays, and medication can be discontinued or switched as necessary.

Kharazmi et al. stated that medical records revealed prodromal symptoms in 86% of cases of bisphosphonate-associated atypical fractures of the femur (n = 18), the most common being pain in the ipsilateral thigh preceding the fracture for weeks or longer [12]. The patients in our series all complained of unexplained thigh pain. We were therefore able to diagnose these three cases at an early stage based on clinical symptoms (i.e., unexplained thigh pain) and X-ray findings (i.e., incomplete fracture with lateral cortical bone hypertrophy). Had we overlooked their symptoms at that point, the patients may have eventually developed complete fractures. This early diagnosis prevented progression to a complete fracture and invasive surgery, allowing patients to achieve early postoperative weight bearing and recovery. Our experience highlights the importance of awareness of patients’ symptoms and making an early diagnosis of atypical femoral fracture, as well as that prophylactic fixation is a reliable method of treatment. Reconstruction nail fixation is the recommended mode of surgical treatment for incomplete atypical femoral fractures [13]. If the femur is so severely curved such that nail insertion is difficult, however, a locking plate can be used instead [14]. Although all our patients had previously undergone surgery on the affected femur, no cases had severely curved shafts; reconstruction nail use was feasible in all cases.

Bisphosphonates may severely suppress bone turnover [1], resulting in secondary calcification and accumulated microdamage that are believed to weaken the bone [2]. We hypothesized that bisphosphonates may delay fracture healing after bisphosphonate-associated incomplete atypical femoral fractures, and that improving bone metabolism may encourage fracture healing. In rat studies, Sloan et al. found that parathyroid hormone may accelerate intracortical bone remodeling induced by microdamage, and that alendronate may delay intracortical bone remodeling during stress fracture repair. Furthermore, parathyroid hormone may be used to facilitate stress fracture repair, whereas bisphosphonates may delay tissue-level repair of stress fractures [15]. In our cases, the time required for synostosis was 15 months in Case 1 (non-teriparatide use), six months in Case 2 (weekly teriparatide use), and five months in Case 3 (daily teriparatide use). These findings suggest that teriparatide administration may shorten the fracture healing period after bisphosphonate-associated incomplete atypical femoral fracture.

This study had several limitations. First, the sample size was small. However, bisphosphonate-associated incomplete atypical femoral fractures are not common and have only recently been reported. Secondly, our discussion of bone metabolic markers and severely suppressed bone turnover was insufficient, since we did not monitor bone metabolic markers. Laboratory tests to measure levels of bone metabolic markers were not performed at all for Case 1. In Case 2, these tests were not performed postoperatively as we were unable to follow-up the patient after bone union was complete (six months postoperatively), meaning that we were unable to compare the levels of bone metabolic markers before and after teriparatide administration. Thus, may be meaningful to measure changes in bone metabolic marker levels over time in future.

This is the first report regarding the treatment of bisphosphonate-associated incomplete atypical femoral fracture by prophylactic intramedullary nail fixation with teriparatide administration (Cases 2 and 3). Our results suggest that prophylactic intramedullary nail fixation with teriparatide administration may be useful for encouraging synostosis and shortening the fracture healing period after bisphosphonate-associated atypical femoral fracture in cases wherein the femoral shaft is not severely curved as to make nailing difficult, and when bone turnover may be suppressed after long-term (more than three years) bisphosphonate use.

## Conclusions

We treated three patients with incomplete atypical femoral fracture, all of whom had been taking bisphosphonates for at least three years. Patients with long-term bisphosphonate use were carefully monitored, and symptoms such as thigh pain should increase suspicion for atypical femoral fracture. The early identification of atypical femoral fracture is key to avoiding complete fracture and difficult surgery. Prophylactic fixation of incomplete atypical femoral fractures is recommended. Teriparatide may be useful for shortening the postoperative time to fracture healing after bisphosphonate-associated incomplete atypical femoral fracture.
